# Chronic Prostatitis/Chronic Pelvic Pain Syndrome Leads to Impaired Semen Parameters, Increased Sperm DNA Fragmentation and Unfavorable Changes of Sperm Protamine mRNA Ratio

**DOI:** 10.3390/ijms22157854

**Published:** 2021-07-23

**Authors:** Elena Berg, Petr Houska, Nils Nesheim, Hans-Christian Schuppe, Adrian Pilatz, Monika Fijak, Marc Manthey, Klaus Steger, Florian Wagenlehner, Undraga Schagdarsurengin

**Affiliations:** 1 Clinic of Urology, Pediatric Urology and Andrology, Justus-Liebig-University, 35392 Giessen, Germany; nesheimn@gmail.com (N.N.); Hans-Christian.Schuppe@derma.med.uni-giessen.de (H.-C.S.); adrian.pilatz@chiru.med.uni-giessen.de (A.P.); marc.p.manthey@med.uni-giessen.de (M.M.); klaus.steger@chiru.med.uni-giessen.de (K.S.); florian.wagenlehner@chiru.med.uni-giessen.de (F.W.); 2 Section Epigenetics of the Urogenital System, Clinic of Urology, Pediatric Urology and Andrology, Justus-Liebig-University, 35392 Giessen, Germany; 3 Department of Urology, Ludwig-Maximilians-University, 80539 Munich, Germany; 4 Institute of Laboratory Medicine and Pathobiochemistry, Molecular Diagnostics, Justus-Liebig-University, 35392 Giessen, Germany; petr.houska@ki.se; 5 ANOVA, Karolinska University Hospital and Karolinska Institutet, 171 77 Stockholm, Sweden; 6 Unit of Reproductive Biology, Institute of Anatomy and Cell Biology, Justus-Liebig-University, 35392 Giessen, Germany; Monika.Fijak@anatomie.med.uni-giessen.de; 7 Section Molecular Andrology, Clinic of Urology, Pediatric Urology and Andrology, Justus-Liebig-University, 35392 Giessen, Germany

**Keywords:** CP/CPPS, infertility, prostate, prostatitis, protamine ratio, sperm DNA damage, sperm DNA fragmentation, sperm quality

## Abstract

Background: Chronic Prostatitis/Chronic Pelvic Pain Syndrome (CP/CPPS) is a frequent disease affecting men of every age and accounting for a great number of consultations at urology departments. Previous studies suggested a negative impact of CP/CPPS on fertility. As increasing attention has been attributed to additional aspects, such as sperm DNA integrity and sperm protein alterations, besides the WHO standard semen analysis when assessing male fertility, in this prospective study, we aimed to further characterize the fertility status in CP/CPPS patients with a focus on these parameters. Methods: Sperm DNA fragmentation measured by sperm chromatin structure assay (SCSA) and protamine 1 to protamine 2 mRNA ratio assessed by RT-qPCR were analyzed along with conventional ejaculate parameters and inflammatory markers in 41 CP/CPPS patients and 22 healthy volunteers. Results: We found significant differences between the groups concerning multiple conventional ejaculate parameters. A significant increase in sperm DNA fragmentation was shown in CP/CPPS patients with association to other sperm parameters. The majority of CP/CPPS patients exhibited protamine mRNA ratios out of the range of regular fertility. Conclusions: This is a pioneering study with a strong practical orientation revealing that CP/CPPS leads to increased sperm DNA damage and changes in sperm protamine levels, emphasizing an unfavorable impact of CP/CPPS on fertility.

## 1. Introduction

Prostatitis syndrome is a frequent condition affecting men of every age with a peak between 36 and 50 years [1]. It accounts for about 8% of all visits to urologists and 1% of consultations at general practitioners [2]. Classified by the NIH into type I Acute bacterial prostatitis, type II Chronic bacterial prostatitis, type III Chronic Prostatitis/Chronic Pelvic Pain Syndrome and type IV Asymptomatic prostatitis, type III accounts for over 90% of all cases, presenting with Prostatitis syndrome and has a high overall prevalence of 10–16% [3,4]. CP/CPPS is subclassified into type IIIA and IIIB depending on the presence of leucocytes in post-prostatic massage urine or prostatic secretions. The condition presents with pain symptoms including pelvic, perineal, scrotal, ejaculatory and pubic pain, urinary symptoms, as well as psychosocial symptoms. Contrary to NIH type I and II, there is no causative pathogen yet detectable in CP/CPPS, and its etiology still remains mostly unknown. As CP/CPPS patients present as a heterogeneous group with a variety of symptoms and symptom-intensity, an individual and multimodal therapeutic approach should be intended. An approach allowing stratification into clinical phenotypes, enabling personalized, phenotypically directed treatment, was developed by the UPOINT(S) classification [5,6]. UPOINTS consists of seven domains: urinary symptoms, psychosocial dysfunction, organ-specific symptoms, infection, neurologic/systemic conditions, tenderness of muscles and sexual dysfunction [6,7,8]. 

As studies have revealed an influence of several diseases of the male urogenital tract on male fertility, the role of CP/CPPS remains a matter of discussion. It has been shown that CP/CPPS is associated with unfavorable effects on semen parameters evaluated by WHO standard semen analysis [9,10]. Recently, when evaluating male fertility, increasing focus has been attributed to additional aspects and parameters besides the WHO standard sperm analysis. One of these parameters is sperm DNA fragmentation, being defined as single and double-strand breaks within sperm DNA. It has a variety of etiologies, which each presumably contributes to multifactorial DNA damage rather than a single responsible factor [11]. Possible pathomechanisms include intrinsic and extrinsic causes, such as abnormal spermatid maturation, abortive apoptosis and oxidative stress caused by reactive oxygen species (ROS) [12]. Moreover, pathologies of the male reproductive system, systemic diseases, iatrogenic and environmental factors are also contributing to these molecular mechanisms [13,14].

Sperm DNA fragmentation can be evaluated by different methods. One of the most commonly used assays is the sperm chromatin structure assay (SCSA) [15], measuring the DNA fragmentation index (DFI), as well as the percentage of high DNA stainability (HDS) [16]. HDS is most likely attributed to immature spermatozoa exhibiting increased amounts of histone-bound DNA or other abnormal proteins resulting in less condensed chromatin [17,18]. 

Multiple studies have shown an association between DNA damage in sperm and male infertility. It has been reported that the chance of spontaneous conception decreases significantly if the DFI is ≥20%, even if patients exhibit conventional semen parameters within the normal range and is close to zero if the DFI exceeds 30–40% [19,20]. Evenson et al., therefore, suggested a clinical threshold of 25% [17]. Sperm DNA fragmentation has also been shown to affect the outcome of assisted reproductive technologies (ART). Patients undergoing intrauterine insemination (IUI) were 7.3 times more likely to achieve pregnancy if the DFI was ≤30% [21], whereas associations with in vitro fertilization (IVF) and intra-cytoplasmatic sperm injection (ICSI) remain controversial.

Protamines (P) are needed to ensure tight compaction of DNA in sperm and replace about 85% of histones during spermatogenesis. Human sperm contains two types of protamines, protamine 1 (P1) and 2 (P2). Varying between different species, protamine content remains constant within one species and occurs in a protamine 1 to protamine 2 ratio of about 1 in human sperm; therefore ratios of 0.8–1.2 are considered normal [22,23]. It has been shown that deviations from this ratio are associated with male subfertility, reduced embryo quality and even unexplained recurrent miscarriages [23,24,25,26]. The P1/P2 mRNA ratio has also been reported to serve as a reliable biomarker for the fertilization potential of men undergoing ART [24]. 

To date, sperm DNA fragmentation, HDS and protamine mRNA ratio to our knowledge have not been extensively analyzed in CP/CPPS patients. In this study, we evaluated these parameters and their relations to conventional semen parameters to further characterize the fertility status of patients with CP/CPPS.

## 2. Results

### 2.1. Basic Semen Parameters, Biochemical Parameters and Inflammatory Parameters

CP/CPPS patients and healthy controls were compared regarding basic semen parameters. The two groups showed significant differences concerning multiple sperm parameters with an overall negative impact in the CP/CPPS group (Table 1). Demographic characteristics, as well as fertility data and lifestyle aspects, for both groups, are shown in Table 2. CP/CPPS patients had significantly decreased ejaculate volumes (*p* < 0.05) and pH (*p* < 0.001), as well as total and progressive motility (*p* < 0.001). Normal morphology and vitality were also presented to be significantly lower (*p* ≤ 0.001). Patients exhibited significantly increased amounts of peroxidase-positive cells (*p* < 0.05) and immature germ cells (*p* < 0.01). Regarding spermatozoa concentration and total spermatozoa per ejaculate, there was no significant difference between the groups, while in the case of total spermatozoa, a considerable tendency of a lower amount within CP/CPPS patients was observable. When looking at biochemical parameters and inflammatory parameters, we did not detect significant differences between the groups in terms of fructose, alpha-glucosidase and elastase. Zinc levels were considerably, however not statistically significant, lower in patients’ ejaculates. Patients exhibited significantly higher levels of IL-8 compared to healthy controls (*p* < 0.01).

### 2.2. Sperm DNA Fragmentation

**Figure 1:**
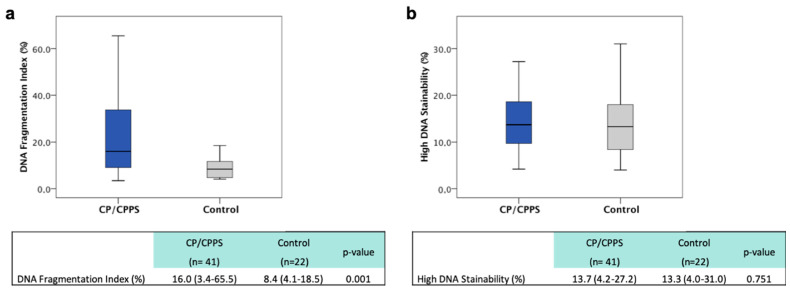
DFI CP/CPPS patients vs. control (**a**); HDS CP/CPPS patients vs. control (**b**).

**Figure A1:**
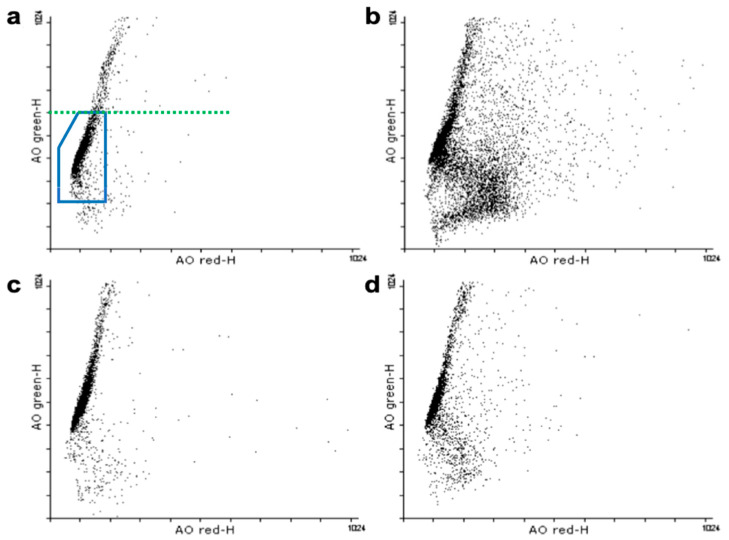
Results of the flowcytometric measurements (SCSA). Detection of green fluorescence is shown on the *y*-axis, detection of red fluorescence on the *x*-axis. The main sperm population is marked blue in (**a**), sperm outside this region on the right exhibit elevated amounts of DNA fragmentation. High DNA stainability can be found above the green line shown in (**a**). (**a**,**b**) represent CP/CPPS patient samples, (**c**,**d**) samples of the control group. (**a**): CP/CPPS with very low amounts of sperm with DNA fragmentation. (**b**): CP/CPPS with high amounts of sperm with DNA fragmentation. (**c**): control with very low amounts of sperm with DNA fragmentation. (**d**): control with high amounts of sperm with DNA fragmentation.

The DNA fragmentation index (DFI) was used to analyze the extent of DNA fragmentation in every sample. Overall, CP/CPPS patients showed significantly increased DFI levels with a median of 16.0% (range 3.4–65.5), compared to the median of the control group (8.4%, range 4.1–18.5) (*p* < 0.01, Figure 1). Both groups exhibited a heterogeneous distribution of the DFI with both very high and very low levels (examples are shown in Figure A1). With regard to reference values, as discussed in the current literature, 36.6% of CP/CPPS patients exhibited a DFI of >25%, while 29.3% of the patients’ samples showed a DFI >30%. None of the control probands exhibited DFI >25%.

**Figure 2:**
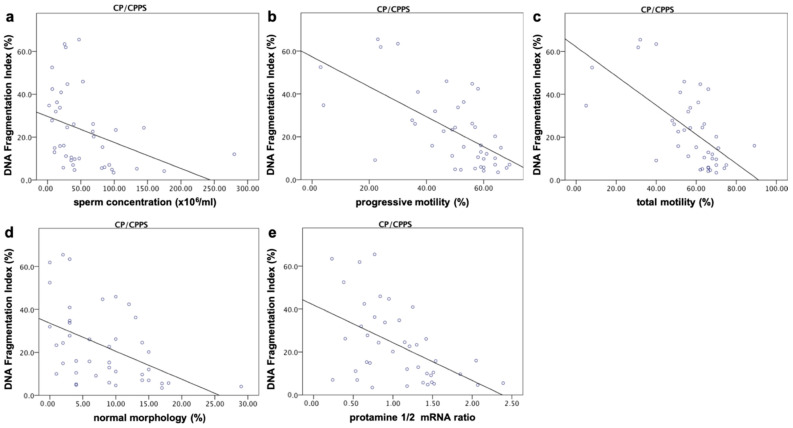
Correlations between DFI and sperm parameters. The DFI showed significant negative correlations with total sperm number (r = −0.320, *p* ≤ 0.05, not shown), sperm concentration (r = −0.508) (**a**), progressive (r = −0.636) and total motility (r = −0.625) (**b**,**c**), normal morphology (r = −0.511) (**d**) as well as the protamine 1/2 mRNA ratio ((r = −0.494) (**e**); for all parameters *p* ≤ 0.001). The DFI was significantly positively associated with sperm head (r = 0.345, *p* ≤ 0.05, not shown) and tail defects (r = 0.621, *p* ≤ 0.001, not shown).

When comparing the DFI in the patient group to semen parameters, significant negative correlations were found with the total number of spermatozoa (r = −0.320, *p* ≤ 0.05), spermatozoa concentration (r = −0.508), total (r = −0.636) and progressive motility (r = −0.625), normal morphology (r = −0.511) as well as the protamine 1/2 mRNA ratio ((r = −0.494); for all parameters *p* ≤ 0.001). The DFI showed significant positive associations with sperm head (r = 0.345, *p* ≤ 0.05) and tail defects (r = 0.621, *p* ≤ 0.001) (Figure 2). 

**Figure 3:**
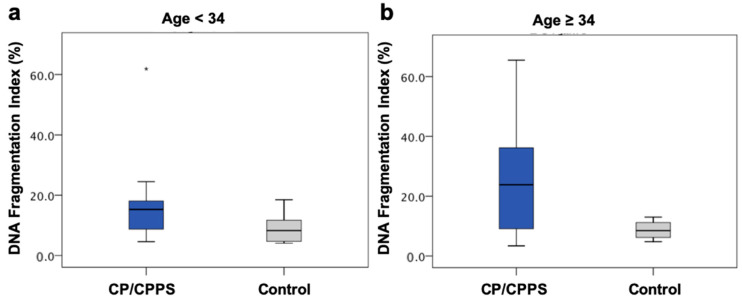
DFI compared after building age-matched groups: (**a**) Age < 34 years DFI: 15.3% (CP/CPPS, *n* = 11, * marks an outlier) vs. 8.3% (Control, *n* = 17), *p* < 0.05. (**b**) Age ≥ 34 years DFI: 23.8% (CP/CPPS, *n* = 30) vs. 8.5% (Control, *n* = 5), *p* = 0.094.

In order to reduce the effect of age on sperm DNA fragmentation, we build age-matched groups. We detected significantly elevated DFI levels in patients when comparing patients and healthy volunteers <34 years (15.3% vs. 8.3%, *p* < 0.05). The older group (≥34 years) showed a clear, though not statistically significant, tendency of increased DNA fragmentation in the CP/CPPS group (23.8% vs. 8.5%, *p* = 0.094) (Figure 3).

### 2.3. High DNA Stainability

The second parameter analyzed by SCSA was high DNA stainability (HDS). To date, there is no reference value for this parameter. When comparing HDS in CP/CPPS patients and healthy volunteers, no significant difference was detected between the groups (*p* > 0.05, Figure 1). 

**Figure 4:**
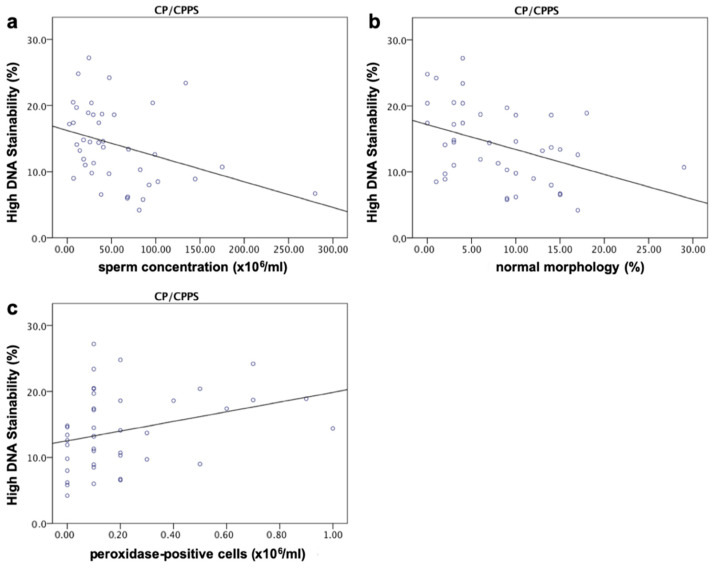
Correlations between HDS and sperm parameters. Significant negative correlations were shown between HDS and total sperm number (r = −0.366, *p* < 0.05, not shown), sperm concentration (r = −0.420, *p* < 0.01) (**a**) and normal morphology (r = −0.399, *p* ≤ 0.01) (**b**). HDS were significantly positively associated with head defects (r = 0.378, *p* < 0.05, not shown) as well as peroxidase-positive cells (r = 0.342, *p* < 0.05) (**c**).

Compared to conventional semen parameters, there was a significant negative correlation between HDS and total sperm number (r = −0.366, *p* < 0.05), sperm concentration (r = −0.420, *p* < 0.01) and normal morphology (r = −0.399, *p* ≤ 0.01). A significant positive association was shown between HDS and head defects (r = 0.378, *p* < 0.05), as well as peroxidase-positive cells (r = 0.342, *p* < 0.05) (Figure 4). There was no significant association between HDS and DFI.

### 2.4. Sperm Protamine mRNA Ratio

**Figure 5:**
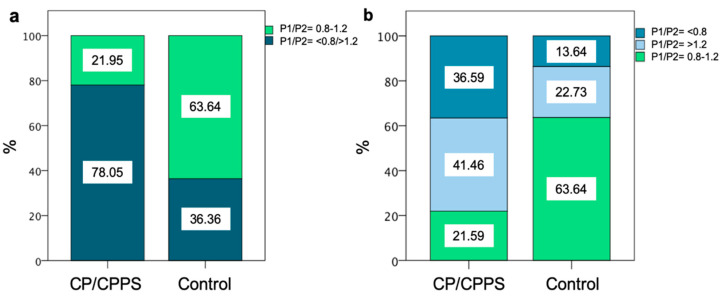
Sperm protamine mRNA ratio in CP/CPPS patients and control group. (**a**) shows the percentage of ratios in (light green) and out of the ratio of 0.8–1.2 (dark green). (**b**) shows the differentiation into protamine ratio below 0.8 (dark blue) and above 1.2 (light blue).

When comparing the median protamine 1/2 mRNA ratio of CP/CPPS patients and healthy controls (1.08 vs. 0.95), no significant difference could be detected. By categorizing into ‘in range’ (0.8–1.2), and ‘out of range’ (<0.8 or >1.2), only 21.95% of patients were ‘in range’, whereas 78.05% exhibited ratios out of this range. In contrast to that, 63.64% of healthy controls had ratios ‘in range’. ‘Out of range’ was further subcategorized into >1.2 and <0.8, showing that 41.46% of CP/CPPS patients had a ratio above 1.2 and 36.59% below 0.8. In the control group, 36.36% presented ratios outside the range, 22.73% above 1.2 and 13.64% below 0.8. Overall, the majority of CP/CPPS patients exhibited protamine 1/2 mRNA ratios out of the range of regular fertility (Figure 5).

Compared to semen parameters, there was a significant negative correlation evident to the DFI (r = −0.494, *p* < 0.01) as well as sperm tail defects (r = −0.357, *p* < 0.05).

### 2.5. DFI, HDS, Protamine mRNA Ratio and Inflammatory Parameters

To evaluate an association between elevated inflammatory parameters and changes in DFI, HDS or the sperm protamine ratio in CP/CPPS patients, we subcategorized the patient group according to the elevation of none (*n* = 14), one (*n* = 22) or more than one (*n* = 5) of the parameters: peroxidase-positive leukocytes (≥1 × 10^6^ per ejaculate), elastase (≥250 ng/mL) and leukocytes, in post-prostatic massage urine. Interestingly there were no significant differences between the parameters depending on the elevation of inflammatory parameters (for all parameters *p* > 0.05). However, the median protamine ratio within the group of more than one elevated inflammatory marker was out of the range of regular fertility.

### 2.6. Basic Semen Parameters, DFI, HDS, Protamine mRNA Ratio and UPOINTS

In this study, we analyzed possible associations between positive UPOINTS domains or certain combinations of positive domains and basic sperm parameters, biochemical and inflammatory parameters, DFI, HDS and protamine mRNA ratio. No significant differences between the groups could be detected in the presence of one particular positive UPOINTS domain (for all parameters *p* > 0.05) or any combination of positive domains (for all parameters *p* > 0.05). The number of positive domains (1–4) was also evaluated for possible associations in regard to the above-named parameters. The parameters did not show significant differences whether patients had one, two, three or four positive UPOINTS domains (for all parameters *p* > 0.05). 

## 3. Discussion

In this pioneering study, we could reveal that CP/CPPS, by leading to increased sperm DNA damage and changes in sperm protamine levels, has an unfavorable impact on male fertility on a molecular level. 

### 3.1. CP/CPPS and Conventional Semen Parameters

Concerning conventional semen parameters in the patient group, we found decreased ejaculate quality in terms of multiple parameters. The results of this study support earlier findings of decreased ejaculate volume in CP/CPPS patients [9]. Ejaculate pH has not routinely been evaluated in earlier studies in CP/CPPS patients. Previous findings analyzing bacterial infections as epididymitis, chronic bacterial prostatitis (CBP) and chronic urethritis suggested significant elevations of seminal pH [28]. Our studies are in line with the results of Schagdarsurengin et al., showing significantly lower seminal pH in CP/CPPS patients’ ejaculate [10]. However, median values of both parameters in our study were still consistent with the WHO reference in both groups, and only a few patients had volumes lying beyond the reference value. Therefore, we assume the observed changes of these parameters might not necessarily have a significant impact on patients’ fertility.

In terms of sperm concentration and total number of spermatozoa per ejaculate, results support the findings of Rusz et al., displaying no significant differences of these parameters in CP/CPPS. In contrast, meta-analyses of Fu et al. and Condorelli et al. found lower sperm concentrations in patients [9,29,30]. Considerably, in our study, every fifth patient exposed sperm concentrations below the WHO reference, supporting results of Schagdarsurengin et al. of oligozoospermia in 21.9% of patients [10]. Even though no significant changes were detected in this study, a negative impact on sperm concentration seems possible.

Sperm motility is an essential factor in the fertilization process. Unfavorable changes concerning this parameter have been observed in different urogenital conditions in the past, such as epididymitis or CBP [9,31]. Reduced normal sperm morphology represents an indicator of testicular stress and can be associated with sperm dysfunction [32]. Negative correlations have been shown between reduced morphology and chromatin condensation, DNA integrity and acrosome reaction [33].

In this study, total and progressive motility, as well as normal morphology, presented to be significantly decreased within patients. While the median was within the WHO reference range in both groups, every tenth patient exhibited asthenozoospermia, every fifth patient teratozoospermia. These results mainly support earlier findings of Condorelli et al. and Fu et al., who observed decreased normal morphology but only decreased progressive motility in CP/CPPS patients, whereas Schagdarsuregin et al. found all three parameters to be negatively affected [9,10,30]. Sperm motility is determined by multiple factors, including changes in the seminal plasma. In the case of urogenital inflammation, the presence of leukocytes and proinflammatory cytokines play a predominant role, amongst others [34,35]. As leukocytes are important sources of ROS, their presence may cause elevated oxidative stress in seminal plasma in case its antioxidative capacity is exceeded. Oxidative stress is able to cause changes in the sperm membrane as well as mitochondrial damage and consecutively decrease sperm motility. Furthermore, cytokines such as IL-6, IL-8 or TNF-α, which can be elevated in CP/CPPS, have previously been shown to negatively affect sperm motility [36,37,38,39].

Overall, the above-named mechanisms might, most likely, be responsible for the changes observed in this study and are also supposedly etiologically relevant in regard to morphology. Especially increased leukocyte activity, consecutive oxidative stress causing loss of membrane integrity and promoting apoptotic processes accompanied by abnormal morphology may play a major role [40]. 

The influence of CP/CPPS on sperm vitality remains controversial. Earlier studies have revealed associations between reduced vitality and CBP but not CP/CPPS; overall Fu et al.’s report states that, most likely, there is no association to this parameter [30]. 

Ultimately, whenever assessing semen parameters, demographic, as well as lifestyle aspects (Table 2), need to be taken into account. 

### 3.2. Biochemical Parameters

The function of the male accessory glands may be affected through urogenital infection. This has been shown for bacterial infection as in CBP, chronic epididymitis or chronic urethritis [28]. Significant decreases of gamma-glutamyltransferase, in the case of inflammatory CP/CPPS, and zinc, as a marker of the secretory function of the prostate, have been revealed in the past. While fructose and alpha-glucosidase, displaying secretory functions of the seminal gland and epididymis respectively, appeared to not be severely affected [9,41]. Our results mostly support these findings, though we observed a tendency towards, however not significant, a decrease in zinc. Zinc has various functions in the prostate, including the preservation of sperm chromatin integrity [9]. With regard to previous studies, an association of CP/CPPS and impaired secretory function of the prostate cannot be excluded. 

### 3.3. CP/CPPS and Sperm DNA Fragmentation

The main aspect of this study was to evaluate the possible impacts of CP/CPPS on sperm DNA fragmentation. We found the DFI to be significantly increased in patients and almost twice as high as in the control group, while more than one-third of patients exhibited values above the determined clinical threshold of 25% [17]. Further, the DFI of 29% of patients exceeded 30%, which is associated with substantially reduced chances of achieving spontaneous pregnancy [17]. Overall, the distribution of the parameter was heterogeneous, with a median DFI within a range that is not necessarily associated with distinctly reduced fertility. Taken together, the assumption of an obligate increase of DNA fragmentation towards fertility-relevant ranges in CP/CPPS cannot be made. Still, considering the significant increase, even in younger patients, and the explicitly high DFI of every third patient, an association to CP/CPPS remains likely. These results support findings concerning other urogenital conditions, as increased DNA fragmentation was found in patients with varicocele or urogenital bacterial infection—e.g., Chlamydia trachomatis [42,43]. The relation of DFI to other sperm parameters or factors such as sperm membrane integrity remains controversial. Several studies found negative correlations with conventional semen parameters, while others could not reproduce these associations [44,45,46]. In this study, we could demonstrate several significant correlations with other sperm parameters. Overall this discrepancy might be caused by the different ways of DNA fragmentation analysis or heterogeneous study populations [44]. While etiologically DNA fragmentation is mainly caused by the three mechanisms named above, ROS and oxidative stress seem to be mainly responsible for DNA damage in sperm. Previous studies found increased ejaculate ROS levels in 25% of subfertile men and significant correlations of DNA strand breaks and 8-hydroxy-2’-deoxyguanosin (8OHdG) as a marker of oxidative stress [11,47]. This might also be one of the main origins of the increased DNA fragmentation found in our study, as previous studies observed elevated levels of oxidative stress and reduced antioxidative capacity in both NIH IIIA and IIIB CP/CPPS [48,49]. 

### 3.4. CP/CPPS and HDS

HDS most likely displays immature sperm with less condensed chromatin, allowing increased stainability by acridine orange. This results from increased amounts of irregular proteins and retained histones following an incomplete histone-protamine-transition [17,18,50,51]. To this point, very few studies have evaluated this parameter, and there is not yet a defined reference value. Evenson et al. found correlations between HDS elevation and reduced fertility rates in vivo when using IUI and IVF and reported HDS related embryo failure at ≥20–25% HDS [17,21]. With no significant differences between patients and controls in this study, both groups exposed moderate median values and maximum values up to 27% and 30%, respectively. The negative correlations of HDS with several basic semen parameters support findings of Virro et al., showing a significantly decreased total number of spermatozoa, motility and morphology when HDS was >15% [52]. Our results provide the first evidence that there is no severely impaired chromatin condensation and no disease-specific elevation of immature sperm containing increased amounts of retained histones in CP/CPPS, while the significance of this parameter and its link to fertility remains limited to date.

### 3.5. CP/CPPS and Sperm Protamine mRNA Ratio

The majority of CP/CPPS patients in our study exhibited P1/P2 mRNA ratios >1.2 and <0.8, meaning an imbalance of these proteins and possibly defective protamination. While the median was within this range in both groups, there was no change towards a specific direction. Aberrant protamine ratios have been associated with male infertility, reduced pregnancy rates, impaired embryo quality and the protamine ratio has also been shown to serve as a marker estimating the fertilizing potential of men in ART programs [24,25,26,53]. While the P1/P2 mRNA ratio in CP/CPPS has not been evaluated to date, previous studies found significant changes in the parameter in other urogenital conditions, such as significant elevations in patients with varicocele or increased rates of aberrant ratios in patients with bacterial infection [43,54]. Our results, for the first time, demonstrate possible changes of this parameter in CP/CPPS and yield a possible negative impact on patients’ fertility. 

### 3.6. Inflammatory Parameters and DFI, HDS, P1/P2 mRNA Ratio

While associations between DNA damage in sperm and leukocytospermia have been reported previously, we did not find significant correlations of the DFI with peroxidase-positive cells in our study [55]. We, therefore, aimed to evaluate possible associations between DFI, HDS, protamine mRNA ratio and an elevation of inflammatory parameters (PPL, elastase, leukocytes in post-prostatic massage urine). We did not find significant differences concerning DFI, HDS, P1/P2 mRNA ratio when comparing patients with no elevated inflammatory parameter to one or more elevated inflammatory parameters, which might be due to the controversially discussed thresholds of inflammatory parameters, such as peroxidase-positive cells in ejaculate or simply displays the interindividual seminal plasma antioxidative capacity. 

## 4. Materials and Methods

### 4.1. Sample Acquisition

The patient group consisted of 41 men with diagnosed NIH type III CP/CPPS (median age 40 years, range 22–62). Both CP/CPPS subgroups, NIH IIIA and IIIB, were included in this prospective study. Exclusion criteria comprised lower urinary tract symptoms (LUTS) with the benign prostate syndrome (BPS), prostate cancer and chronic epididymitis/orchitis. The control group consisted of 22 healthy volunteers above 18 years of age without preexisting urological conditions (median age 29.5 years, range 20–41). All participants attended outpatient clinic consultations at the Department of Urology, Pediatric Urology and Andrology at Justus-Liebig-Universität Giessen and their clinical presentation was assessed according to the present guidelines [56,57]. Participants underwent a structured urological and andrological examination. Their symptoms were evaluated using NIH-CPSI [58], and each patient was classified by UPOINTS [5,59]. Participants provided blood samples, pre- and post-prostatic massage urine, as well as ejaculate samples. Bacterial infection was excluded by culture and molecular diagnostics in urines and ejaculate, including multiplex PCR for C. trachomatis, N. gonorrhoeae, M. genitalium, M. hominis, U. urealyticum, U. parvum, T. vaginalis and in culture-negative samples 16SrRNA analysis [60,61]. 

All patients and healthy controls were informed about the study and provided their written consent. The study was approved by the Ethics Commission of the Medical Faculty of JLU Giessen (ethical vote, AZ 55/13).

### 4.2. Semen Analysis

Ejaculates were obtained after 2–7 days of sexual abstinence and analyzed within 1 h of collection according to the WHO 2010 recommendations [27]. Briefly, after complete liquefaction and thorough mixing for representative sampling of homogenous aliquots, sperm motility was analyzed by means of phase-contrast microscopy using wet preparations obtained from native semen samples. Sperm concentration was determined in a Neubauer improved hemocytometer. For the evaluation of sperm morphology, semen smears were prepared, air-dried and subjected to Shorr staining; normal sperm morphology was defined on the basis of strict criteria. Each parameter was measured in duplicate, analyzing at least 200 spermatozoa in each aliquot. Moreover, 2 × 100 µL aliquots of liquefied native ejaculate were extracted and stored at −80 °C for sperm DNA fragmentation measurements. Peroxidase-positive cells were analyzed to quantify the number of seminal leukocytes in the ejaculate (LeucoScreen, FertiPro). Ejaculates were centrifuged (5 min at 13,000 rpm). Seminal plasma was extracted, and fructose, alpha-glucosidase (both in-house assays, [62]), zinc (Zinc Assay, Wako Chemicals), polymorpho-nuclear elastase (PMN Elastase ELISA, Demeditec Diagnostics, Kiel, Germany) and interleukin-8 (Human IL-8 ELISA Kit, BD Biosciences) were measured by standardized methods. The remaining ejaculate pellets were stored for further processing (RNA isolation) at −80 °C. All measurements were conducted from one sample per patient/control to exclude intraindividual differences. 

### 4.3. Sperm Chromatin Structure Assay (SCSA)

A sperm chromatin structure assay was carried out based on earlier protocols [15]. Acridine orange (AO) equilibration buffer was prepared, consisting of 200 µL acid detergent solution and 600 µL acridine orange staining solution, followed by the equilibration of the flow cytometer (MACSQuant^®^ Analyzer 10, Miltenyi Biotec) for 8 min. A reference sample with a defined DFI was measured in duplicate at the beginning of sample measurement and following every five samples to ensure constant conditions of every measurement. Frozen semen samples were thawed on ice and diluted with TNE buffer (1.42 g Tris-HCl, 7.98 g NaCl, 0.33 g Na2EDTA; dissolved in 900 mL ddH20) to 2 × 10^6^ sperm cells/mL. A total of 100 µL of each sample were mixed with 200 µL acid detergent solution, and a stopwatch was immediately started. After exactly 30 s, 600 µL of AO staining solution was added to stain the spermatozoa. The sample was then placed into the flow cytometer, and data acquisition was initiated at 3 min on the stopwatch, followed by the analysis of at least 5000 cells, while the maximum flow did not exceed 300 cells/s. Data analysis was conducted by Flowing Software 2 (Centre of Biotechnology, Turku). The DNA fragmentation index (DFI) was calculated by DFI = red fluorescence/total (red + green) fluorescence. Histograms exhibiting the distribution of the whole sperm population, dependent on each DFI, were generated to determine the percentage of sperm with increased DFI (%DFI). As a second parameter, the population of sperm with high DNA stainability (%HDS) was analyzed after defining a cut-off value exposing excessive stainability. Exemplary results of SCSA measurements are shown in Figure A1.

### 4.4. RNA Isolation, cDNA Synthesis and RT-qPCR

Ejaculate samples were thawed on ice, washed with 500 µL PBS (phosphate-buffered saline; 5 PBS tablets in 1 L ddH20) twice and then pelletized for 5 min at 13,000 rpm. The pellets were resuspended in 150 µL peqGOLD Trifast™ (Peqlab) and treated with TissueLyser LT (Qiagen) for 5 min (50 oscillations per s, metal ball added). After the addition of 100 µL chloroform, the samples were incubated at room temperature for 5 min and centrifuged for 10 min at 4 °C, 13,000 rpm. The aqueous phase was used for RNA isolation, mixed with 1 volume isopropanol and 5 µL glycogen prior to incubation at −20 °C. RNA pellets were then centrifuged for 30 min at 4 °C, 13,000 rpm and washed twice with 500 µL 75% EtOH in diethylpyrocarbonate (DEPC) containing water. The samples were treated with DNAse I prior to RT-qPCR.

RNA concentrations were measured using NanoDrop 1000 (Thermo Scientific). RNA samples were reverse transcribed in cDNA using 500 ng RNA with MMLV-RT (Promega) in a volume of 40 µL. cDNA samples were purified with a QIAquick Nucleotide Removal Kit (Qiagen) according to the manufacturers’ protocol. Quantitative PCR was performed in duplicates with IQ™ SYBR Green Supermix (Bio-Rad) and CFX96 Real-Time System (Bio-Rad). PCR conditions were 95 °C for 5 min, 40 cycles of 95 °C for 30 s, 60 °C for 30 s, 72 °C for 30 s followed by 72 °C for 5 min, 55 °C for 5 s and 95 °C for 30 s. Primer sequences (Eurofins) included PRM-1 5′AAGTCGCAGACGAAGGAGG3′ (forward primer), PRM-1 5′ATCTCGGTCTGTACCTGGGG3′ (reverse primer) resulting in an 80 bp PCR product and PRM-2 5′AAGACGCTCCTGCAGGCAC3′ (forward primer), PRM-2 5′GCCTTCTGCATGTTCTCTTCCT3′ (reverse primer) resulting in a 71 bp PCR product. Human testes cDNA served as the positive control (Human Testis Total RNA, Clontech). ‘No template’ controls containing ddH2O were used as negative controls. RT-qPCR efficiency was evaluated based on standard curves of known protamine 1 and protamine 2 quantities. The protamine 1/2 mRNA ratio was determined as described previously [24].

### 4.5. Statistical Analysis

Statistical analysis was carried out with SPSS Statistics 24.0 (IBM). Median values and ranges (minimum–maximum) are given for age, basic semen parameters, biochemical parameters, SCSA data and protamine mRNA ratio. Non-parametric variables were compared using the Mann–Whitney U-test (2-sided). Correlations between parameters were calculated using Spearman’s rank correlation. *p* values < 0.05 were considered statistically significant.

## 5. Conclusions 

In summary, the results of this study demonstrate significant differences concerning multiple conventional semen parameters in CP/CPPS patients compared to healthy men. The data reveal, for the first time, that CP/CPPS patients exhibit increased sperm DNA fragmentation and mainly aberrant protamine ratios, which overall emphasizes an unfavorable impact on fertility on a molecular level. The observed changes were not necessarily linked to elevated inflammation markers but the presence of CP/CPPS itself. Further studies are needed to identify patient subgroups affected by detrimental impairments in particular, as well as possible effects of therapeutical approaches on fertility.

## Figures and Tables

**Table 1:** Basic semen parameters and biochemical ejaculate parameters of CP/CPPS patients and healthy controls (median values and ranges are given).

Parameter	CP/CPPS	Control	*p*-Value	WHO 2010 [27] Lower Reference Limit */Consensus Threshold Values
Volume (mL)	2.3 (0.6–7.4)	3.5 (1.1–5.0)	**0.046**	1.5 *
pH	7.8 (7.2–9.0)	8.3 (7.8–8.7)	**<0.001**	7.2 *
Total sperm number (×10^6^/ejaculate)	81.2 (4.3–896.0)	132.1 (42.0–725.2)	0.139	39 *
Sperm concentration (×10^6^/mL)	38.3 (2.4–280.0)	44.8 (12.0–148.0)	0.545	15 *
Total motility (%)	62.0 (5.0–89.0)	75.5 (55.0–88.0)	**<0.001**	40 *
Progressive motility (%)	53.0 (3.0–69.0)	64.5 (42.0–84.0)	**<0.001**	32 *
Immotile sperm (%)	37.0 (11.0–95.0)	24.5 (12.0–45.0)	**<0.001**	N/A
Normal morphology (%)	8.0 (0.0–29.0)	14.0 (4.0–31.0)	**0.001**	4 *
Head defects (%)	79.0 (51.0–100.0)	69.0 (48.0–91.0)	**0.003**	N/A
Mid-piece defects (%)	4.0 (1.0–22.0)	53.0 (33.0–74.0)	**<0.001**	N/A
Tail defects (%)	51.0 (20.0–89.0)	21.0 (7.0–36.0)	**<0.001**	N/A
Vitality (%)	61.0 (34.0–82.0)	86.5 (73.0–97.0)	**<0.001**	58 *
Peroxidase-positive cells (×10^6^/mL)	0.1 (0.0–1.0)	0.0 (0.0–1.5)	**0.011**	<1
Immature germ cells (×10^6^/mL)	0.56 (0.0–22.4)	0.0 (0.0–2.29)	**0.005**	N/A
Fructose (µmol/ejaculate)	42.2 (9.1–158.0)	45.8 (9.8–116.6)	0.462	≥13
Glucosidase (mU/ejaculate)	40.6 (4.1–242.0)	40.4 (19.6–134.8)	0.713	≥20
Zinc (µmol/ejaculate)	6.6 (1.3–40.8)	10.3 (4.0–24.5)	0.066	≥2.4
Elastase (ng/mL)	37 (10–1191)	31.5 (10–415)	0.674	<250
IL-8 (pg/mL)	3503 (956–21,524)	1679.5 (458–20,226)	**0.004**	N/A

* lower reference limit.

**Table 2:** Demographic characteristics, fertility data and lifestyle aspects of CP/CPPS patients and control group including number of children, couples currently trying to conceive, fertility treatment in terms of assisted reproductive technology (ART), smoking, alcohol and drug use.

			CP/CPPS (%) (*n* = 41)	Control (%) (*n* = 22)
**Median age in years (range)**			40 (22–62)	29.5 (20–41)
**Children**	0		41.5	86.4
	1		14.6	4.5
	2		19.5	9.1
	≥3		14.6	0
	no data		9.8	**-**
**Currently trying to** **conceive**	yes		12.2	22.7
	no		41.5	77.3
	no data		46.3	-
**ART**	yes		2.4	0
	no		43.9	100
	no data		53.7	-
**Smoking**	yes		22.0	9.1
	no		68.3	90.9
	no data		9.8	-
**Alcohol**	yes	rarely	14.6	31.8
		occasionally	22	45.5
		frequently	14.6	22.7
	no		36.6	0
	no data		12.2	-
**Drug use**	yes		2.4	9.1
	no		46.3	90.9
	no data		51.2	-

## Data Availability

The data presented in this study are available in the article.
